# The Influence of LepR Tyrosine Site Mutations on Mouse Ovary Development and Related Gene Expression Changes

**DOI:** 10.1371/journal.pone.0141800

**Published:** 2015-11-03

**Authors:** Xiaoyu Tu, Zhichao Kuang, Xia Gong, Yan Shi, Lin Yu, Huijuan Shi, Jian Wang, Zhaogui Sun

**Affiliations:** 1 Shanghai Medical College, Institute of Reproduction and Development, Fudan University, Shanghai, P. R. China; 2 Key Laboratory of Contraceptive Drugs and Devices of National Population and Family Planning Commission of China, Shanghai Institute of Planned Parenthood Research, Shanghai, P. R. China; 3 Department of Food Science, Shanghai Business School, Shanghai, P. R. China; Institute of Zoology, Chinese Academy of Sciences, CHINA

## Abstract

Leptin exerts many biological functions, such as in metabolism and reproduction, through binding to and activating the leptin receptor, LepRb, which is expressed in many regions of the brain. To better understand the roles of LepR downstream signaling pathways, Y123F mice, which expressed mutant leptin receptors with phenylalanine (F) substituted for three tyrosines (Y) (Tyr985, Tyr1077 and Tyr1138), were generated. The body weight and abdominal fat deposits of Y123F homozygous mice (HOM) were higher than those of wild-type mice (WT). HOM ovaries were atrophic and the follicles developed abnormally; however, the HOM ovaries did not exhibit polycystic phenotypes. Moreover, Y123F HOM adults had no estrous cycle and the blood estrogen concentration remained stable at a low level below detection limit of 5 pg/ml. LepR expression in HOM ovaries was higher than in WT ovaries. Using cDNA Microarrays, the mRNA expressions of 41 genes were increased, and 100 were decreased in HOM *vs*. WT ovaries, and many signaling pathways were evaluated to be involved significantly. The expressions of 19 genes were validated by real-time quantitative PCR, most of which were consistent with the microarray results. Thus, Y123F HOM mice were suggested as a new animal model of PCOS for research that mainly emphasizes metabolic disorders and anovulation, but not the polycystic phenotype. Meanwhile, using the model, we found that JAK-STAT and hormone biosynthesis pathways were involved in the follicular development and ovulation disorders caused by LepR deficiency in ovaries, although we could not exclude indirect actions from the brain.

## Introduction

Leptin, the 16 kDa adipocyte hormone product of the ob gene [1], was originally thought to be an anti-obesity hormone. However, in addition to controlling energy balance and body weight as a messenger of energy stores to the brain [2], extensive research has shown that leptin is also a crucial hormone/cytokine in other physiological processes, such as inflammation, glucose homeostasis [3], bone metabolism [4], immune function [5], and most importantly, reproduction [6].

The leptin receptor is the product of alternative mRNA splicing of the db gene, and has six isoforms (LepRa, b, c, d, e, and f) divided into three classes: long, short, and secretory [7]. All isoforms have an extracellular leptin-binding domain, but only the long form, LepRb, contains a full-length intracellular domain mediating leptin signaling [8]. Leptin exerts its biological action by binding to and activating LepRb, which is expressed in many brain regions, as well as certain peripheral tissues; however, the physiological function of peripheral LepRb remains to be determined [9,10,11,12]. LepRb-deficient db/db mice display obese phenotypes similar to those of leptin-deficient ob/ob mice [9,13]. In addition, LepRb-deficient mice also exhibit other metabolic and reproduction abnormalities [14].

Accordingly, using transgenic technology, Jiang Lei et al. generated knock-in lines of mice expressing mutant leptin receptors with phenylalanine (F) substituted for all three tyrosines (Y) residues within exon 18 of LepRb (Tyr985, Tyr1077 and Tyr1138; denoted Y123F) [15]. Y123F mice also manifested obesity, hyperphagia, hyperleptinemia, hyperinsulinemia and impaired glucose tolerance, but less severely than db/db mice [16].

For many years, leptin/LepR-deficient mice, such as db/db mice, have been used as animal model to study polycystic ovary syndrome (PCOS) [16], which is widely acknowledged as the most frequent female endocrine disorder, with a variety of etiological factors and clinic manifestations. PCOS affects 5%~10% of women, causing infertility caused by dysfunctional follicular maturation and ovulation, distinctive polycystic ovaries and hyperandrogenism, together with metabolic abnormalities, including obesity, hyperinsulinism and an increased risk of type 2 diabetes [17,18]. These metabolic and reproductive characteristics are somewhat similar to those of db/db mice; however, some PCOS patients suffer from moderate metabolic disorders as well as infertility, which led us to consider that the Y123F mice might represent a new PCOS model.

However, the abnormal reproduction characteristics of Y123F mice and the gene expression changes in the ovary remain unclear. Whether leptin signaling can directly act on the ovary and how this leads to ovarian pathological changes has yet to be determined. Here, for the first time, we investigated the morphological changes of Y123F murine ovaries compared with those of wild-type (WT) mice, and characterized the follicular developments and identified related gene expression profiling and signaling pathways using a cDNA microarray to explore the potential mechanistic links between LepR tyrosine mutations-induced obesity and reproduction abnormalities. We further evaluated whether Y123F mice could be a new animal model of PCOS.

## Materials and Methods

### Animals

Adult heterozygous Y123F mice were kindly provided by Dr Shi Huijuan at Shanghai Institute of Planned Parenthood Research, Shanghai, China. They were backcrossed to the WT C57BL/6 mice (purchased from Shanghai Laboratory Animal Research Center, Shanghai, China) for at least three generations to yield heterozygous Y123F mice in the C57BL/6 background, which were then inbred to produce homozygous Y123F mice and WT littermates. Mice were genotyped by PCR analysis of tail-tip DNA at 4 weeks of age. All mice were fed ad libitum with a standard diet and maintained in a temperature-and light-controlled room (22–24°C with 55%–65% relative humidity and a 12/12 hours light/dark cycle). The study was approved by the Animal Ethics Committee at Shanghai Institute of Planned Parenthood Research. Animals were cared for in accordance with the principles of the Guide to the Care and Use of Experimental Animals.

### Phenotypic studies

Mice were weighed at the 4^th^ week, 8^th^ week and 12^th^ week after birth. At 12 weeks of age, all mice were euthanized by decapitation. Blood samples were collected and centrifuged, and the serum was stored at −20°C for further analysis. Ovaries were excised, cleaned of fat, weighed, and frozen at −80°C. Abdominal fat deposits were dissected and weighed using an analytical balance (Shanghai ShangTian Precision Instrument Co., Shanghai, China). The amount of estrogen was quantified using a commercial ELISA kit (Shanghai Hu Shang Bio Technology Co., Shanghai, China), according to the manufacturer's instructions.

### Estrous cycle analysis

At 10 weeks of age, the estrous cycles of mice were monitored by vaginal smears between 8:00 and 9:00 AM for 12 consecutive days. Cells that were detached from the vaginal epithelium were removed with a cotton swab saturated with normal saline (NaCl, 0.9%), and then placed on a clean glass slide to create well-proportioned tracks of smears. The slides were then stained with Giemsa and examined under a Leica DM750 microscope (Leica, Nussloch, Germany) at 10× and 40× magnifications. Stages were assessed based on vaginal cytology [19]. A proestrus smear consisted predominantly of nucleated cornified cells; an estrus smear consisted primarily of anucleated cornified cells; a metestrus smear consisted of an equal proportion of leukocytes, cornified and nucleated epithelial cells; and a diestrus smear consisted primarily of leukocytes. A 4 to 5 day estrous cycle was determined to be a regular cycle, and a cycle duration of >5 days or <4 days was considered to be an irregular cycle [20]. Those displaying constant estrus, intermittent regularly, and intermittent irregular estrous cycles were also defined as irregular cycling [21].

### Hematoxylin and eosin (HE) staining

Ovaries were fixed for 72h in 4% paraformaldehyde in PBS (pH 7.2). After serial dehydratation steps, the samples were embedded in paraffin and serially sectioned at a thickness of 5μm. Sections were deparaffinized, hydrated, stained with HE, and analyzed under a Leica DM750 microscope (Leica).

### Immunohistochemistry for the Leptin Receptor

Sections of paraffin-embedded ovaries (5μm) from each mouse were collected on glass slides, then deparaffinized and hydrated. Antigen retrieval was achieved by microwaving the slides in 0.01M sodium citrate solution (pH 6.0) for 15min, cooling to room temperature and washing three times in phosphate buffered saline (PBS) each for 5 min. After washing, the slides were blocked with 3% hydrogen peroxide in PBS for 10 min to quench endogenous peroxidase activity. The nonspecific background was eliminated by blocking with 10% lamb serum in PBS for 1 h at room temperature. The slides were then incubated with rabbit polyclonal anti-LepR antibody (Boster, Wuhan, China) at a 1:50 dilution in 10% lamb serum in PBS and incubated overnight at 4°C. After washing with PBS, the slides were incubated for 1 h at room temperature with biotinylated goat anti-rabbit IgG antibody (Invitrogen, Carlsbad, CA, USA) diluted 1:200 in 10% lamb serum in PBS. Immunoreactive signals were detected using streptavidin-HRP and VECTOR Nova RED Peroxidase (HRP) Substrate Kit (Vectorlabs, Burlingame, CA, USA) at room temperature. Slides were counterstained with hematoxylin. A negative control was performed by omitting the primary antibody incubation step. Immunostaining was observed using a Nikon Eclipse 50i microscope (Nikon, Tokyo, Japan) and captured by NIS-Element F Software.

### Western blotting analysis for the Leptin Receptor

Total proteins were extracted from whole ovaries on ice in lysis buffer (Beyotime, Shanghai, China). Lysates were homogenized using a homogenizer and centrifuged at 12 000 × g for 15 min at 4°C and the protein concentrations were measured using a BCA Protein Assay Kit (CWBIO, Beijing, China). Approximately 10 μg of protein extracts were mixed with 1× loading buffer to denature them and before separation by electrophoresis on an SDS polyacrylamide gel for 1.5h. The electrophoresed proteins were then transferred to 0.45-μm nitrocellulose membrane (ImmobilionTM-NC Millipore Corporation, MMAS, USA) for 2.0h. The membranes were then blocked with 7% nonfat dry milk powder (NFDMP) in Tris-buffered saline (TBS: 2 mM Tris-HCl, 15 mM NaCl, pH 7.4), containing 0.1% Tween-20 (TBST) for 1h at room temperature. The membranes were incubated overnight at 4°C with rabbit polyclonal anti-LepR antibody (Boster) (dilution 1/300) in TBST with 3.5% NFDMP. After several washes in TBST, they were incubated for 1h at room temperature with a horseradish peroxidase-conjugated anti-rabbit IgG (dilution 1/2000) in TBST with 3.5% NFDMP. After washing again in TBST, the signals were detected by SuperSignal^™^ West Dura Extended Duration Substrate (Thermo Fisher Scientific, MMAS, USA) and analyzed using the FluorChem Gel documentation system (Tanon, Beijing, China). Band intensities were analyzed by Image J software. GAPDH was used as a loading control, using an antibody against GAPDH from Proteintech Group, USA. The results were obtained from four independent western blotting experiments.

### Gene profiling using Illumina Microarrays

Three experimental sets comprising six ovaries of WT mice and Y123F mice were used for microarray analysis. Total RNA was isolated from mouse ovaries using the Trizol Total RNA isolation reagent (Biostar Genechip inc, Shanghai, China), according to the manufacturer’s instructions. RNA concentration was measured by UV spectrophotometry, and OD 260/280nm ratios between 1.7 and 2.0 were obtained for all RNA samples. The quality of the total RNA was determined by electrophoresis. The RNA of each group of three mice was evenly mixed, and 5μg of the mixed RNA sample was used to synthesize cDNA. The RNA sample was converted into double-stranded cDNA, and then cRNA was synthesized and amplified cRNA using the Illumina^®^TotalPrep RNA Amplification Kit, according to the manufacturer’s instructions. Illumina Microarrays with the Mouse whole-Genome Hybridize 6-Sample BeadChip were used, and an Illumina BeadStation 500 System (Illumina, San Diego, CA, USA) was used to plot and analyze the microarray data. Probe-level data analysis was performed on the Illumina Beadstudio Application using the robust multichip analysis method. Normalization and differential analysis algorithms were used to determine the target genes, with detection p-value <0.01 and the relative change in the diffscore of >20 or <-20. The LepR regulated genes in ovaries that were screened out were indicated as signal fold differences of Y123F compared with WT controls. The processed data were exported and presented as tables. The microarray CEL files, normalized data and experimental information have been deposited in the Gene Expression Omnibus, and are available by the accession number GSE73590. Using pathway analysis software in the Kyoto Encyclopedia of Genes and Genomes (KEGG) public database, genes were screened according to the KEGG classification for the significant pathways involved.

### Quantitative real-time PCR (qPCR)

QPCR was performed to quantify the expressions of *Gapdh*, *Cfd*, *Ovgp1*, *Cited4*, *C3*, *Hsd17b7*, *Ugt1a10*, *Acsl4*, *Adora1*, *Cish*, *Timp1*, *Prlr*, *Nupr1*, *Kit*, *Serpina5*, *Fabp3*, *Insl3*, *Lep*, *Serpina3c* and *Gpha2* in the ovary. The primer sets are shown in Table 1. Total RNA was extracted from each tissue using Trizol (Invitrogen). RNA purity and quantity was confirmed using a NanoDrop ASP-3700 spectrophotometer (ACTGene, Piscataway, NJ, USA), OD 260/280nm ratios between 1.8 and 2.0 were obtained for all RNA samples. Total RNA from each sample was reverse transcribed into cDNA using a standard oligo-dT RT protocol with a ReverTra Ace qPCR RT Kit (Toyobo, Osaka, Japan). QPCR reactions were carried out in a 10-ul reaction volume with SYBR Green I Master Mix reagent (Toyobo, Osaka, Japan) and 5 pmol of the specific primer pairs shown in Table 1. The PCR thermal cycling conditions were 95°C for 10 min for polymerase activation and the initial denaturation step, followed by 40 cycles with denaturation at 95°C for 30s, annealing at 60°C for 30s and extension at 72°C for 30s. A melting curve analysis was recorded at the end of the amplification to evaluate the absence of contaminants or primer dimers. Each set of qPCR reactions was repeated three times, and the levels of gene expression were expressed in the form of 2^-ΔΔCt^.

**Table 1:** Primers used in quantitative real-time PCR.

Gene	Accession number	Primer sequences (5′–3′)
Cfd	NM _001291915.1	Upstream:ACTGCATGGATGGAGTGACG
		Downstream:CGTATTGCAAGGGTAGGGGT
Ovgp1	NM _007696.2	Upstream: TGCTGTCGGCTGCTGTCTCT
		Downstream:ATCTGCGGGTGTCCCAAGCT
Cited4	NM _019563.2	Upstream: TGCCAGATGACAGTTGGGTC
		Downstream:CGGAGAGATGGCTCCCTAGA
C3	NM _009778.3	Upstream: TGGAGGTCAAGGCTGCTGTCT
		Downstream:CTGGCACTTGGTCGCTAAGGT
Hsd17b7	NM _010476.3	Upstream: CTGTGACACCGTACAACGGA
		Downstream: GCTCGGGTGATCCGATTTCT
Ugt1a10	NM _201641.2	Upstream: AACTGTCTCCAGGGGAAGCC
		Downstream:TTCGATGGTCTAGTTCCGGTG
Acsl4	NM _001033600.1	Upstream: AGAACGCTGGTGGAATCCTACG
		Downstream:TCAGCAACAGCAAGCAGACCAT
Adora1	NM _001008533.3	Upstream: ACAGGAGGGAACGGAGAGGTT
		Downstream: TGTGGATTGTCGGCTGGAGGT
Cish	NM _009895.3	Upstream: GCCAGACAGACAGGAGCCAGAA
		Downstream:GCAGGAAACGGACACAAGGGAG
Timp1	NM _001044384.1	Upstream: GGCATCTGGCATCCTCTTGT
		Downstream: GCTGGTATAAGGTGGTCTCGT
Prlr	NM _001253781.1	Upstream: ATCTTTCCACCAGTTCCGGG
		Downstream: TGGTGGTGGAACCCATTTTGA
Nupr1	NM _019738.1	Upstream: CAATACCAACCGCCCTAGCC
		Downstream: GGCCTAGGTCCTGCTTACAAC
Kit	NM _001122733.1	Upstream: TGCGTGTGGGTGAGTTGTGTTG
		Downstream: AGGTCCTTCGGCACTTCACTCC
Serpina5	NM _172953.3	Upstream: CCACCCATGCTGACTTGTCT
		Downstream: GGTCAGCAGAAAGGGTCTGG
Fabp3	NM _010174.1	Upstream: GTGACAGCAGATGACCGGAA
		Downstream: CTCACCACACTGCCATGAGT
Insl3	NM _013564.7	Upstream: ACCGCTGCTGTCTTACTGGCT
		Downstream: AGGAGGTGGGCACAGGTCAT
Lep	NM _008493.3	Upstream: AAATGTGCTGGAGACCCCTG
		Downstream: GGGTGAAGCCCAGGAATGAA
Serpina3c	NM _008458.2	Upstream: AGCAGACTTCCAGCAGCCTCT
		Downstream: TGGGCACCTTCACAGACCTCT
Gpha2	NM _130453.3	Upstream: CCAGTGGCTATCGGCACAACA
		Downstream: GTAGCGGGAGAAACGGCACAT
GAPDH	NM _001289726.1	Upstream: GTGAAGGTCGGTGTGAACGGATT
		Downstream: GGTCTCGCTCCTGGAAGATGGT

### Statistical Analysis

All data are presented as Mean ± SD. Statistical analyses were performed with unpaired two-tailed Student’s t test; a p-value < 0.05 was considered statistically significant.

## Results

### Phenotypic evaluation of two mouse genotypes

**Fig 1:**
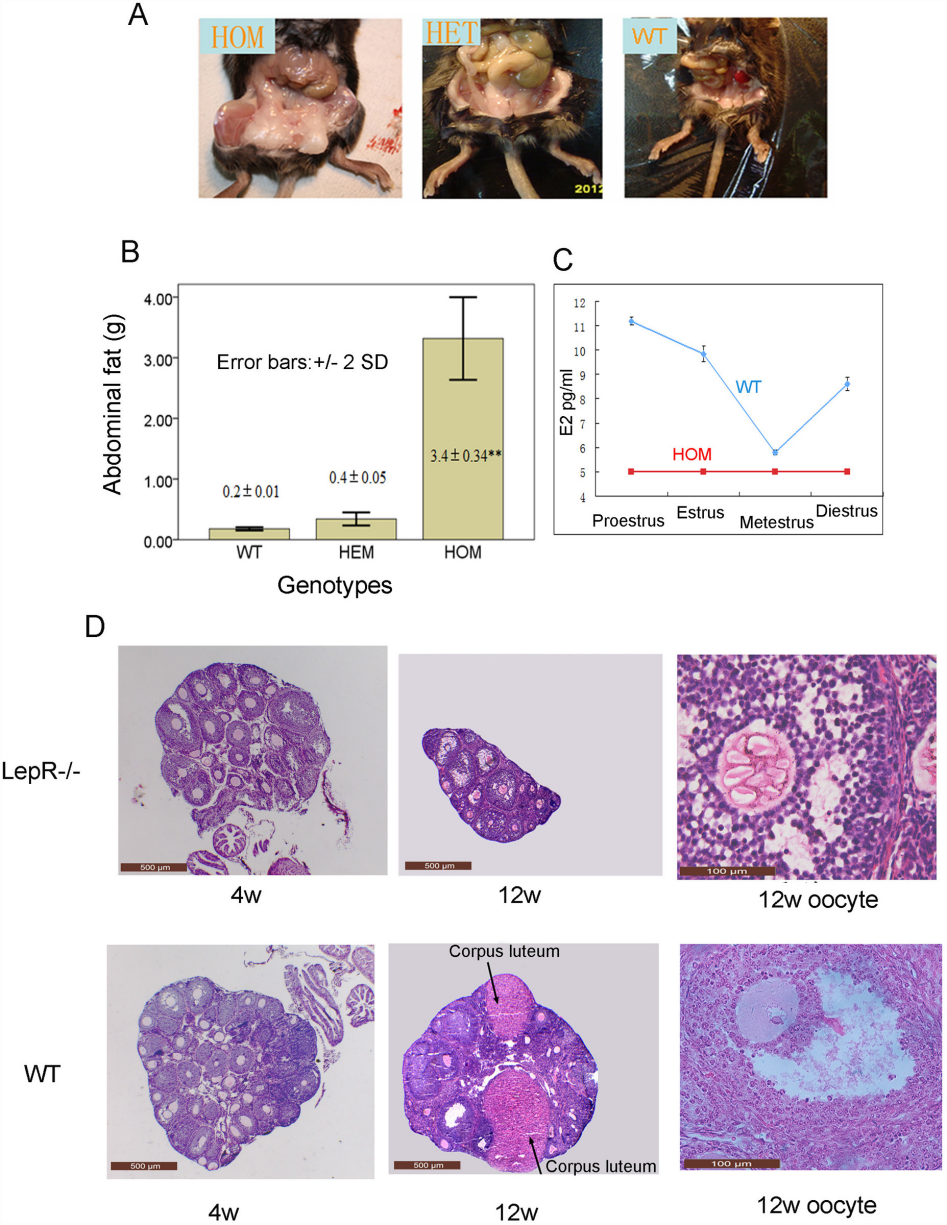
The phenotypic analysis of the LepR mutant mouse Y123F. Panel A: Images of mouse peritoneal anatomy showing the abdominal fat accumulation at postnatal week 12. HOM, homozygous mutation; HET, heterozygous mutation; WT, wild type. Panel B: The comparative statistics of abdominal fat accumulation among different genotypes of mice at 12 weeks of age, as shown in Panel A. Panel C: Concentrations of estrogen throughout the estrous cycle of two mouse genotypes at postnatal week 12. Panel D: Ovarian morphology of the HOM and WT mice at postnatal week 4 and 12 In the top row, from left to right (in order) are ovarian sections of mutant homozygous mice at the age of 4 and 12 weeks (4×10 magnification), and a local enlargement picture of the ovarian section of 12w (10×10 magnification), which includes a typical follicle to show the characteristics of the contained oocyte. In the bottom row, in parallel, are the corresponding controls of the WT.

According to the results of PCR analysis of tail-tip DNA and 2% gel electrophoresis, for the Y123 mice two bands at 400bp and 450bp represented the heterozygous genotype; one band at 450bp represented the homozygous genotype; and one band at 400bp represented the WT (figure not shown). We focused on two groups: homozygous genotype (HOM) and the WT. Compared with WT mice, the body weight of HOM mice began to increase significantly from 4 weeks of age. The difference in body weight was statistically significant between the two groups at the 4th week (p <0.05), 8th week (p<0.01) and 12th week (p<0.01). Similarly, the difference in abdominal fat deposits was significant at the 12th week (p<0.01; higher in the HOM *vs*. WT), while the ovarian weights were lower in the HOM mice than in the WT mice (p<0.01) (Table 2, Fig 1A and 1B).

**Table 2:** Body weight (BW), abdominal fat deposits and ovarian weight in HOM and WT groups (mean ± SD, g for BW and fat deposits, mg for ovary weight).

genotype	4th week (BW)	8th week (BW)	12th week (BW)	12th week (fat deposits)	12th week (ovary weight)
HOM(n = 8)	10.3 ±0.75 *	28.4 ±1.28**	38.0 ±1.38**	3.4 ±0.34**	7.5 ±3.17**
WT (n = 8)	7.8 ±0.67	19.1 ±0.65	20.7 ±0.57	0.2 ±0.01	17.0 ±3.10

Note: Asterisks indicate significant differences in the same column (*: P < 0.05, **:P < 0.01).

### The estrous cycle and corresponding estrogen level in two mouse genotypes

Monitoring 10 of each mouse genotype by vaginal smears for 12 consecutive days at the age of 10 weeks, showed that the WT mice exhibited regular and complete estrous cycle lasting for 4 to 5 day, specifically with four distinct stages: predominantly nucleated oval cornified cells and occasionally cornified cells were seen in proestrus smears; predominantly anucleated irregular cornified epithelial cells and occasionally nucleated cells were seen in estrus smears; leukocytes, irregular cornified and columnar epithelial cells were seen in metestrus smears; leukocytes were seen primarily in diestrus smears. By contrast, the HOM mice lacked estrus cycles: their cell morphology did not change and anucleated cornified epithelial cells were always observed during the 12 days (images not shown). At postnatal week 12, mice at different stages of the estrous cycle were sacrificed to collect blood. Immediately following blood collection, the mice were examined again by observing vaginal smears to confirm their estrous cycle stages. The concentration of estrogen of WT mice fluctuated throughout the estrous cycle, reaching a peak at proestrus and the lowest level at metestrus, whereas the estrogen concentration of HOM mice remained stable at a low level (below 5pg/ml, the lower limit of detection), which were all recorded as 5pg/ml for convenience (Fig 1C).

### Ovarian morphology of two mouse genotypes

At 4 weeks, no significant differencee between the ovaries and follicles of HOM and WT mice were observed by HE staining. At postnatal week 12, HE staining demonstrated different ovarian morphology between the two groups. Developing follicles and corpora lutea were observed in WT ovaries, which indicated normal ovulation function. Although developing follicles could be observed in HOM ovaries, ovulation was not observed. In addition, HOM ovaries were atrophic and immature; the oocytes of some tertiary follicles developed abnormally because they were fragmented and had thickened zona pellucida, as it can be seen in the magnified picture on the right in Fig 1D.

### LepR expression changes of Y123F female mouse ovaries

**Fig 2:**
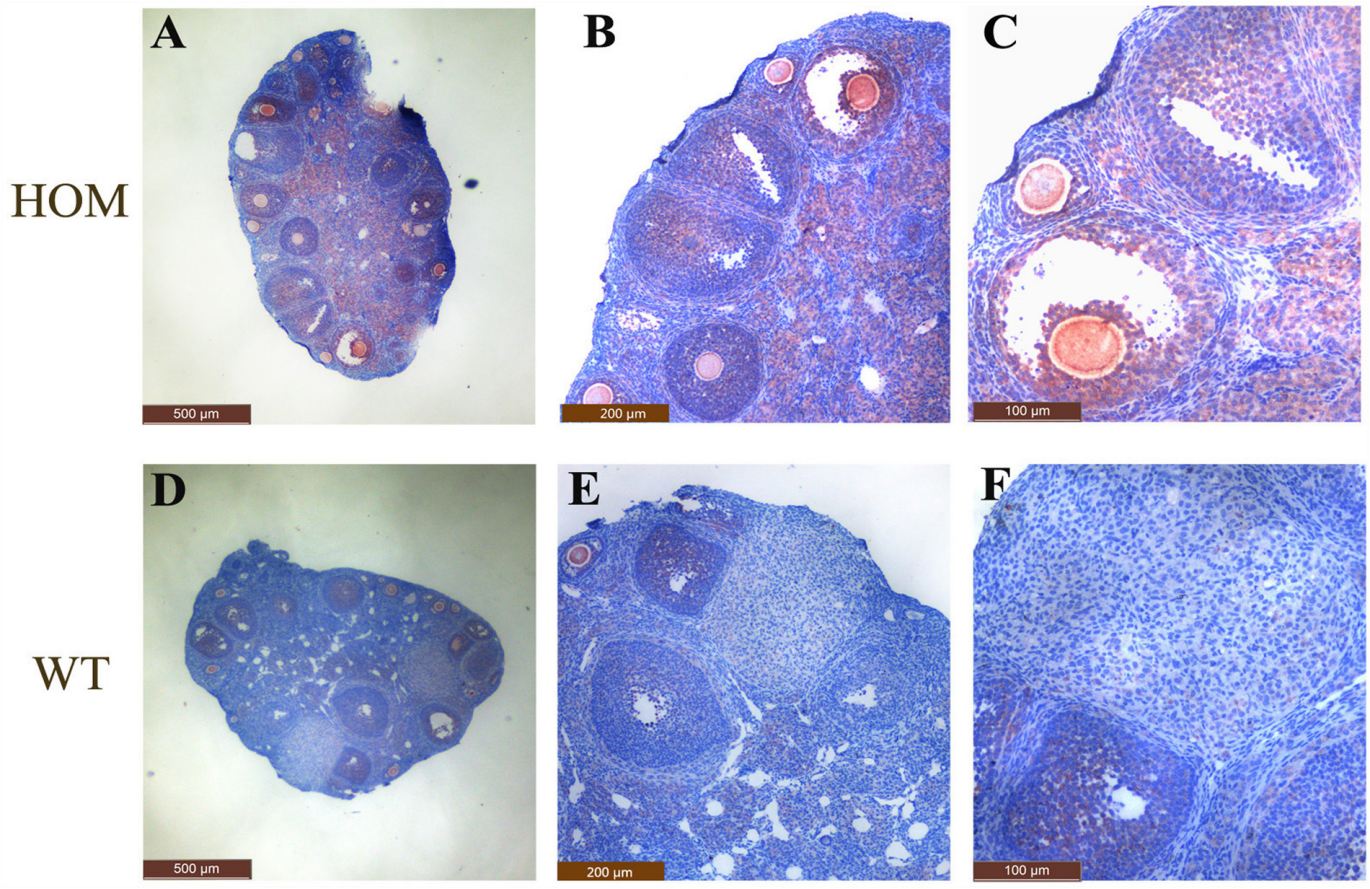
Immunohistochemical detection of LepR in HOM and WT ovaries. Panel A, B, C: LepR expression in HOM ovaries observed at 4×10 magnification, 10×10 magnification and, 20×10 magnification, respectively. Panel D, E, F: LepR expression in WT ovaries observed at 4×10 magnification, 10×10 magnification and 20×10 magnification, respectively. LepR expression was mainly detected at the surfaces of theca cells and granulosa cells of follicles. The total expression level of LepR in WT ovaries was significantly lower than in HOM ovaries.

**Fig 3:**
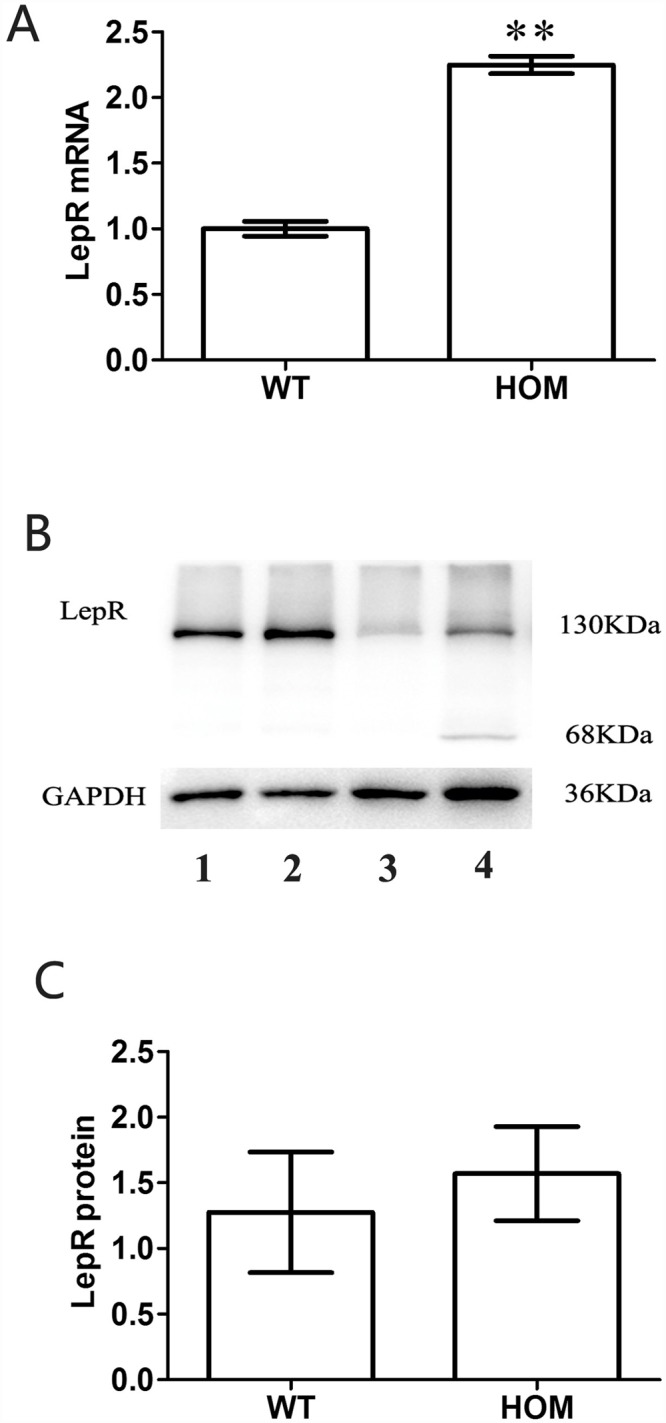
Detection of LepR mRNA and protein levels in ovarian tissue extracts of LepR Y123F mutated Homozygous (HOM) and wild mice (WT). Panel A, Relative ovarian mRNA expressions of *LepR* in HOM and WT ovaries (n = 3). All data were normalized by WT, and bar values are presented as mean ±SD. **p<0.01. The mRNA expression level of *LepR* in HOM ovaries was significantly higher than in WT ovaries. Panel B. LepR protein expression level detection by western blotting. Upper row is LepR, and the lower row is the internal control of GAPDH. Lanes 1 and 2 were loaded with ovary samples of WT and HOM, respectively; and lanes 3 and 4 with kidney and brain samples. Panel C. Densitometric analysis of western blotting detection of LepR. Data are represented as mean ±SD of measurements from four independent experiments. No statistical difference was found.

To examine the expression of LepR in the ovaries of the two genotypes, immunohistochemistry and western Blotting were performed. As shown in Fig 2, immunohistochemistry showed that the total expression level of LepR in WT ovaries was significantly lower than in the HOM group and LepR was distributed mainly in granulose cells of the secondary follicle stage. Meanwhile, the mRNA level of *LepR* in HOM ovarian tissues was significantly higher than in WT, as shown in Fig 3A. Western blotting showed that LepRb was significant expressed in the mouse ovary compared with GAPDH, using brain as a positive control and kidney as a negative control; however, expression level of LepR was not statistically different between the HOM and WT group. Moreover, other LepR isoforms were also expressed in brain as well as LepRb, whereas ovary mainly expressed LepRb. (Fig 3B and 3C)

### Gene profiling by applying Illumina chips

According to the Illumina method, the ratio of HOM and WT gene expression levels were compared, and genes with statistically significant differences was identified. The original data were uploaded into GEO library, with login number of GSE73590. The expression of 41 genes was increased in HOM *vs*. WT mouse ovaries, and the expression of 100 genes was decreased (S1 Table). The significant genes were grouped into different categories that were related to many signaling pathways, such as GSH metabolism pathways, including microsomal glutathione S-transferase 2 (Mgst2, HOM/WT = 0.17), isocitrate dehydrogenase 1 (NADP+), soluble (Idh1, HOM/WT = 0.51); MAPK signaling pathway including growth arrest and DNA-damage-inducible 45 alpha (Gadd45a, HOM/WT = 0.38), mitogen-activated protein kinase-activated protein kinase 3 (Mapkapk3, HOM/WT = 0.36); Jak-STAT signaling pathway including prolactin receptor (Prlr, HOM/WT = 0.46), leptin (Lep, HOM/WT = 4.46), cytokine inducible SH2-containing protein (Cish, HOM/WT = 0.28); GnRH signaling pathway including, calcium channel, voltage-dependent, L type, alpha 1D subunit (Cacna1d, HOM/WT = 3.10), Jun oncogene (Jun, HOM/WT = 0.35); PPARγ signaling pathway including fatty acid binding protein 3, muscle and heart (Fabp3, HOM/WT = 2.36), acyl-CoA synthetase long-chain family member 4 (Acsl4, HOM/WT = 0.236); hormone biosynthesis pathway including hydroxysteroid (17-beta) dehydrogenase 7(Hsd17b7, HOM/WT = 0.21), UDP glycosyltransferase 1 family, and polypeptide A10 (Ugt1a10, HOM/WT = 0.18) (Table 3).

**Table 3:** Pathways involved in LepR gene site mutation of Y123F mice.

KEGG pathways associated with LepR	Identity
Glutathione metabolism	480
Jak-STAT signaling pathway	4630
MAPK signaling pathway	4010
Peroxisome	4146
Complement and coagulation cascades	4610
Cytokine-cytokine receptor interaction	4060
Steroid hormone biosynthesis	140
Bile secretion	4976
Adipocytokine signaling pathway	4920
PPAR signaling pathway	3320

### Validation of gene expression

QPCR was used to validate significant expression changes of selected genes. Nineteen genes were selected according to possible related KEGG pathways: *Kit*, *Serpina5*, *Fabp3*, I*nsl3*, *Lep* and *Gpha2* were up regulated in the microarrays and in qPCR validation. *Cfd*, *Cited4*, *C3*, *Hsd17b7*, *Ugt1a10*, *Acsl4*, *Adora1*, *Cish*, *Timp1*, *Prlr* and *Nupr1* were all down regulated in qPCR detection and in the microarray experiments. Only *Serpina5*, *Fabp3* and *C3* showed non-significant expression changes in the qPCR analysis. Only two genes, *Serpina3c* and *Ovgp1*, had the opposite results in the microarray experiments compared with the qPCR validation (Table 4).

**Table 4:** The expression differences of 19 selected genes from cDNA microarrays used for qPCR validation.

Gene	HOM/WT	
	microarrays	qPCR
*acsl*	0.23	0.61 ±0.03**
*adora*	0.27	0.41 ±0.06**
*C3*	0.20	0.81 ±0.11
*Cfd*	0.04	0.50 ±0.02**
*Cish*	0.28	0.70 ±0.04**
*Cited4*	0.13	0.39 ±0.03**
*Fabp3*	2.34	1.14 ±0.08
*Gpha2*	6.08	2.08 ±0.26**
*Hsd17b7*	0.21	0.39 ±0.01**
*Insl3*	3.30	3.14 ±0.24**
*Kit*	2.17	2.91 ±0.04**
*Lep*	4.46	12.70 ±0.83**
*Nupr1*	0.46	0.73 ±0.04**
*Ovgp1*	0.08	2.97 ±0.39**
*Prlr*	0.42	0.12 ±0.02**
*Serpina3c*	5.69	0.61 ±0.03**
*Serpina5*	5.07	1.18 ±0.08
*Timp1*	0.30	0.33 ±0.02**
*Ugt1a10*	0.18	0.58 ±0.02*

QPCR data indicated as mean ± SD, where * indicates p<0.05, and ** indicates p<0.01.

## Discussion

In the present study, we investigated the effects of LepR tyrosine mutations on mouse phenotypic characteristics, mainly associated with reproduction. In accordance with previous studies [15,22], Y123F HOM mice displayed early-onset obesity (from 4 weeks of age) and they had higher BWs and more abdominal fat deposits than their WT littermates, though these differences were less pronounced than in db/db mice [15], fully illustrating the importance of leptin and LepR in adiposity metabolism and energy balance. Meanwhile, Y123F HOM mice showed atrophic ovaries, acyclicity, anovulation, increased follicular atresia and infertility, and, especially, hormone changes, including a significant decrease in serum E2 levels, all of which were similar to db/db mice [23,24]. However, polycystic ovaries were not observed in either model [16]. Therefore, Y123F HOM mice can only mimic some characteristics of PCOS, as do db/db mice. Considering that some PCOS patients present with moderate metabolic disorders but with significant reproductive disorders, Y123F HOM mice might represent a new and more appropriate animal model of PCOS than db/db mouse in some cases.

Consequently, we detected LepR expression in the ovaries of HOM and WT mice. The mRNA level of *LepR* in Y123F mice ovaries was significantly higher than in WT ovaries, which might be caused by physiological compensation. The abnormal LepR cannot perform its role effectively; the organism increases its mRNA transcription level through positive feedback regulation to try to compensate for the deficiencies of LepR. Intriguingly, there was a statistical difference at the level of mRNA but not at the level of protein, which might reflect changes in protein degradation of abnormal LepR proteins. However, immunohistochemistry showed that the total expression level of LepR in HOM ovaries was significantly higher than in the WT, presumably because LepR is distributed mainly in granulosa cells of the secondary follicle stage. HOM ovaries contained more secondary follicles than the WT because of disordered follicular development. The identification of LepR expression in ovaries suggested that leptin might have a direct effect on the ovary in HOM mice; however, this does not exclude its indirect actions from the brain. There is no clear consensus on the predominant function of leptin in this particular facet of ovarian physiology, because leptin has been demonstrated to either inhibit ovarian steroid synthesis [25,26,27] or to stimulate aromatase activity and estrogen secretion [28]. In addition, LepR tyrosine mutations-induced obesity also contributed to the reproductive abnormality of Y123F mice, because obesity can lead to decreased LH pulse amplitude and decreased excretion of progesterone metabolites. This may affect ovarian follicular steroidogenesis, leading to abnormal oocyte recruitment and poor oocyte quality and/or altered endometrial development, which could affect the function of the corpus luteum in the luteal phase [29].

To identify other genes and signaling pathway that might be involved in the reproductive abnormality of Y123F mouse ovaries, we used high-density cDNA microarrays to analyze Y123F mouse ovaries and qPCR to verify the microarray data. We identified a group of genes involved in corresponding pathways that changed dramatically in Y123F mouse ovaries.

CISH: CISH (cytokine-inducible SH2 protein) is a member of the SOCS (suppressor of cytokine signaling) family, which comprises eight proteins: SOCS1-7 and CISH. These proteins have similar structures and function as inhibitors of cytokine signaling [30]. All family members contain a central SH2 domain for binding to phosphorylated tyrosines, and a C-terminal ‘SOCS-box’ domain for directing targeted proteins for proteasomal degradation [31]. CISH interacts with LepR by specifically binding to the conserved Y985 and Y1077 motifs in the cytosolic domain. CISH functions through competition with STAT binding at the receptor recruitment site, which may involve inhibition of recruitment of downstream signaling moieties [32]. CISH may also influence growth through regulation of LepR signaling, probably depending on STAT5, since it is activated in different cell types upon leptin stimulation *in vitro* through the Y1077 and Y1138 positions in LepR [33]. In addition, Matsumoto et al observed that CISH itself is a target of STAT5, because they isolated and characterized the promoter region of the *CISH* gene, which contained MGF boxes that are recognized by STAT5 [34]. In our study, *CISH* gene expression decreased dramatically in Y123F mouse ovaries because of the abnormal LepR, indicating the possible involvement of the JAK-STAT pathway in ovulation and growth defects.

Hsd17b7: 17b-Hydroxysteroid dehydrogenase type 7 (17HSD type7) is a membrane-associated reductive enzyme belonging to the 17b-Hydroxysteroid dehydrogenases (17HSDs) family, which has the ability to interconvert keto- and hydroxy-groups on position C17 of the steroid backbone, thereby controlling the biological action of hormones [35]. Rodent Hsd17b7 catalyzed the conversion of estrone (E1) to estradiol (E2) and was particularly abundantly expressed in the ovaries of pregnant animals [36]. Besides, mouse Hsd17b7 messenger RNA was obviously and exclusively expressed in a proportion of corpora lutea, indicating Hsd17b7 was the enzyme needed for E2 biosynthesis in the corpora lutea [37]. Moreover, the corpora lutea, which were well vascularized and mainly consisted of normal glandular epithelial cells with nuclei equal in size and pronounced cytosol, expressed more Hsd17b7, indicating a characteristic of an active corpora lutea. By comparison, corpora lutea lacking Hsd17b7 exhibited signs of regression, and a few small corpora lutea showed negative for Hsd17b7 due to luteolysis and involution [37]. Given this, in our study, the decreased expression of Hsd17b7 in Y123F mouse ovaries could contribute to anovulation and the low level of estrogen.

Ugt1a10 (UDP-glucuronosyltransferases (UGTs), comprising two families, UGT1 and UGT2, are a class of membrane-bound glycoproteins that biotransform a large number of endogenous and exogenous compounds produced by Phase II enzymes [38]. UGTs from both families are directly involved in the biotransformation of estradiol (E2), and the 4-hydroxylated catechol-estrogens (4-OH-CEs) by catalyzing glucuronidation reactions [39]. The activated estrogen-ER complex can interact with cofactors that modify ER’s action, either by enhancing or inhibiting target gene transcription [40]. Ugt1a10 was identified a novel estrogen-regulated target gene because UGT1A10 mRNA expression was stimulated, and then decreased, by E2 in a dose- and time-dependent manner when acting on its corresponding estradiol receptors (ER) [41]. Therefore, in our study, the low level of E2 may be attributed to the observation that UGT1A10 gene expression was decreased in Y123F mice ovaries, as shown by both microarray and qPCR.

Kit: Tyrosine-protein kinase Kit, also known as proto-oncogene c-Kit, is a receptor tyrosine kinase protein encoded by the *KIT* gene in humans [42]. Kit forms a dimer that activates its intrinsic tyrosine kinase activity, which in turn phosphorylates and activates signal transduction molecules to propagate the signal in the cell. Previous studies demonstrated the expression profiles of the kit ligand and c-Kit within the human ovary, which suggested that they could be functionally relevant to reproduction, though little is known about their specific roles [43,44]. Meanwhile, studies using animal models have revealed the functions of the kit ligand and c-Kit in oocytes and follicle development through interactions between granulosa cell-derived kit ligand and oocytes and theca cell-derived c-Kit. This included the establishment of primordial germ cells within the ovary, primordial follicle activation, oocyte survival and growth, granulosa cell proliferation and theca cell recruitment [45]. In our study, the kit gene exhibited increased expression in Y123F HOM mouse ovaries, indicating its possible involvement in the early stage of follicular development.

In summary, our data suggested that Y123F mice might be a new animal model of PCOS for research that mainly emphasizes metabolic disorders and anovulation, but not the polycystic phenotype. The identification of LepR expression in the ovary suggested that leptin might have a direct effect on the ovary in HOM mice, although this does not exclude its indirect actions from the brain. Microarray and qPCR analyses of Y123F mouse ovaries indicated that the LepR defects affected many pathways, including the JAK-STAT signaling pathway and hormone biosynthesis pathway, which are involved in ovary development and ovulation. Although not all the involved genes in Y123F mouse ovaries could be discussed and verified, the roles of the related genes and pathways identified herein could be deduced by the mutation-induced changes in their expressions. The detailed mechanisms of the interactions among these genes will be further studied to illuminate the relationship between LepR and reproduction, and between obesity and reproduction, especially in the ovary, which might help to clarify the mechanism of PCOS.

## Supporting Information

S1 Table Gene expression differences between Y123F and WT by Illumina Microarrays. S1 Table gives gene lists with significant changes of mRNA levels in ovaries of the mouse strain (denoted Y123F) with artificially mutated leptin receptors, with phenylalanine (F) substitution for all 3 tyrosine (Y) residues within exon 18 of LepRb of Tyr985, Tyr1077 and Tyr1138, with wild types of the same litter as the control. Y123F mice also manifested obesity, hyperphagia, hyperleptinemia, hyperinsulinemia and impairment in glucose tolerance. Young mice subjected to cDNA microarray analysis, LepR/WT indicated the Y123F signal value/control signal value. Ovarian tissues of 12 weeks old Y123F and Control mice was taken from 6 ovaries of 6 mice respectively, and then mixed into RNA pools for the DNA microarray analysis by United Gene Technology Co Ltd (Shanghai). Mouse WG-6 v2.0 chip (microarray chips, Illumina) was used as the mouse whole genome expression profiling bead chip. The original data were uploaded into GEO library (GSE73590).
(DOCX)
