# Supplementary material for: The Influence of LepR Tyrosine Site Mutations on Mouse Ovary Development and Related Gene Expression Changes
Source: PLoS One. 2015 Nov 3;10(11):e0141800. doi: 10.1371/journal.pone.0141800 (PMC4631549; doi:10.1371/journal.pone.0141800)
Supplement: S1 Table — gives gene lists with significant changes of mRNA levels in ovaries of the mouse strain (denoted Y123F) with artificially mutated leptin receptors, with phenylalanine (F) substitution for all 3 tyrosine (Y) residues within exon 18 of LepRb of Tyr985, Tyr1077 and Tyr1138, with wild types of the same litter as the control. Y123F mice also manifested obesity, hyperphagia, hyperleptinemia, hyperinsulinemia and impairment in glucose tolerance. Young mice subjected to cDNA microarray analysis, LepR/WT indicated the Y123F signal value/control signal value. Ovarian tissues of 12 weeks old Y123F and Control mice was taken from 6 ovaries of 6 mice respectively, and then mixed into RNA pools for the DNA microarray analysis by United Gene Technology Co Ltd (Shanghai). Mouse WG-6 v2.0 chip (microarray chips, Illumina) was used as the mouse whole genome expression profiling bead chip. The original data were uploaded into GEO library (GSE73590). (DOCX) [file pone.0141800.s001.docx]

**S1 Table. Gene expression differences between Y123F and WT by Illumina Microarrays.** S1 Table gives gene lists with significant changes of mRNA levels in ovaries of the mouse strain (denoted Y123F) with artificially mutated leptin receptors, with phenylalanine (F) substitution for all 3 tyrosine (Y) residues within exon 18 of LepRb of Tyr985, Tyr1077 and Tyr1138, with wild types of the same litter as the control. Y123F mice also manifested obesity, hyperphagia, hyperleptinemia, hyperinsulinemia and impairment in glucose tolerance. Young mice subjected to cDNA microarray analysis, LepR/WT indicated the Y123F signal value/control signal value. Ovarian tissues of 12 weeks old Y123F and Control mice was taken from 6 ovaries of 6 mice respectively, and then mixed into RNA pools for the DNA microarray analysis by United Gene Technology Co Ltd (Shanghai). Mouse WG-6 v2.0 chip (microarray chips, Illumina) was used as the mouse whole genome expression profiling bead chip. The original data were uploaded into GEO library (GSE73590).

**Gene expression differences between Y123F and WT by Illumina Microarrays**

| **Number** | **SYMBOL** | **LepR/WT** | | **DEFINITION** |  |
| --- | --- | --- | --- | --- | --- |
| 1 | Cemip | 0.015 | | cell migration inducing protein, hyaluronan binding |  |
| 2 | Abcb1b | 0.017 | | ATP-binding cassette, sub-family B, member 1B |  |
| 3 | Cfd | 0.042 | | complement factor D |  |
| 4 | S3-12 | 0.050 | | plasma membrane associated protein, S3-12 |  |
| 5 | S100a6 | 0.068 | | S100 calcium binding protein A6 |  |
| 6 | Ovgp1 | 0.076 | | oviductal glycoprotein 1 |  |
| 7 | Tnc | 0.087 | | tenascin C |  |
| 8 | Tmem178 | 0.107 | | transmembrane protein 178 |  |
| 9 | Adrb2 | 0.117 | | adrenergic receptor, beta 2 |  |
| 10 | Cryba4 | 0.124 | | crystallin, beta A4 |  |
| 11 | Cited4 | 0.129 | | Cbp/p300-interacting transactivator, with Glu/Asp-rich carboxy-terminal domain, 4 |  |
| 12 | Clstn3 | 0.131 | | calsyntenin 3 |  |
| 13 | Ltf | 0.138 | | lactotransferrin |  |
| 14 | Myo7a | 0.139 | | myosin VIIa |  |
| 15 | Myl1 | 0.139 | | myosin, light polypeptide 1 |  |
| 16 | Sgk1 | 0.142 | | serum/glucocorticoid regulated kinase 1 |  |
| 17 | Emb | 0.158 | | embigin |  |
| 18 | Rab27a | 0.159 | | RAB27A, member RAS oncogene family |  |
| 19 | Stx11 | 0.167 | | PREDICTED: syntaxin 11 |  |
| 20 | Gm2a | 0.169 | | GM2 ganglioside activator protein |  |
| 21 | Mgst2 | 0.174 | | microsomal glutathione S-transferase 2 |  |
| 22 | Ugt1a10 | 0.177 | | UDP glycosyltransferase 1 family, polypeptide A10 |  |
| 23 | Avpi1 | 0.185 | | arginine vasopressin-induced 1 |  |
| 24 | C3 | 0.196 | | complement component 3 |  |
| 25 | Hsd17b7 | 0.207 | | hydroxysteroid (17-beta) dehydrogenase 7 |  |
| 26 | Sbsn | 0.216 | | suprabasin |  |
| 27 | Rasgrp1 | 0.222 | | RAS guanyl releasing protein 1 |  |
| 28 | Lrrn1 | 0.222 | | leucine rich repeat protein 1, neuronal |  |
| 29 | Ltbp1 | 0.225 | | latent transforming growth factor beta binding protein 1 , transcript variant 2 |  |
| 30 | Scx | 0.225 | | scleraxis |  |
| 31 | Ctsc | 0.231 | | cathepsin C |  |
| 32 | Fbxl22 | 0.233 | | F-box and leucine-rich repeat protein 22 |  |
| 33 | Acsl4 | 0.234 | | acyl-CoA synthetase long-chain family member 4 , transcript variant 3 |  |
| 34 | Ramp1 | 0.236 | | receptor activity modifying protein 1 |  |
| 35 | Sh3gl2 | 0.251 | | SH3-domain GRB2-like 2 |  |
| 36 | Lgmn | 0.254 | | legumain |  |
| 37 | Osbpl3 | 0.257 | | oxysterol binding protein-like 3 |  |
| 38 | Ephx2 | 0.268 | | epoxide hydrolase 2, cytoplasmic |  |
| 39 | Adora1 | 0.269 | | adenosine A1 receptor , transcript variant 1 |  |
| 40 | Il10rb | 0.272 | | interleukin 10 receptor, beta |  |
| 41 | Gpx3 | 0.272 | | glutathione peroxidase 3, transcript variant 2 |  |
| 42 | Hist2h2aa1 | 0.272 | | histone cluster 2, H2aa1 |  |
| 43 | Mapkapk3 | 0.274 | | mitogen-activated protein kinase-activated protein kinase 3 |  |
| 44 | Nr1h4 | 0.276 | | nuclear receptor subfamily 1, group H, member 4 |  |
| 45 | Cish | 0.276 | | cytokine inducible SH2-containing protein |  |
| 46 | Prune2 | 0.281 | | Prune2 prune homolog 2 |  |
| 47 | Iglc2 | 0.289 | | Iglc2 - immunoglobulin lambda constant 2 |  |
| 48 | Mrap | 0.290 | | melanocortin 2 receptor accessory protein |  |
| 49 | Mbp | 0.292 | | myelin basic protein , transcript variant 8 |  |
| 50 | Cyp11a | 0.295 | | cytochrome P450, family 11, subfamily a, polypeptide 1 |  |
| 51 | Timp1 | 0.298 | | tissue inhibitor of metalloproteinase 1, transcript variant 2 |  |
| 52 | Star | 0.313 | | steroidogenic acute regulatory protein |  |
| 53 | Tnfrsf12a | 0.315 | | tumor necrosis factor receptor superfamily, member 12a |  |
| 54 | Wisp1 | 0.320 | | WNT1 inducible signaling pathway protein 1 |  |
| 55 | Ces3 | 0.323 | | carboxylesterase 3 |  |
| 56 | Acss2 | 0.325 | | acyl-CoA synthetase short-chain family member 2 |  |
| 57 | Kiaa0513 | 0.331 | | RIKEN cDNA 6430548M08 gene |  |
| 58 | Txndc2 | 0.331 | | thioredoxin domain containing 2 |  |
| 59 | Pnpo | 0.334 | | pyridoxine 5'-phosphate oxidase |  |
| 60 | Dpysl4 | 0.337 | | dihydropyrimidinase-like 4 |  |
| 61 | Lgals3 | 0.337 | | lectin, galactose binding, soluble 3 |  |
| 62 | Cyp4f14 | 0.339 | | cytochrome P450, family 4, subfamily f, polypeptide 14 |  |
| 63 | Jun | 0.348 | | Jun oncogene |  |
| 64 | Ecm1 | 0.349 | | extracellular matrix protein 1 |  |
| 65 | Slc46a3 | 0.354 | | solute carrier family 46, member 3 |  |
| 66 | Slc25a30 | 0.354 | | solute carrier family 25, member 30 |  |
| 67 | Hist2h2aa2 | 0.354 | | histone cluster 2, H2aa2 |  |
| 68 | Acss1 | 0.361 | | acyl-CoA synthetase short-chain family member 1 |  |
| 69 | Cdkn1a | 0.364 | | cyclin-dependent kinase inhibitor 1A |  |
| 70 | Homer2 | 0.366 | | homer homolog 2 |  |
| 71 | Stard5 | 0.369 | | StAR-related lipid transfer domain containing 5 |  |
| 72 | Gadd45a | 0.376 | | growth arrest and DNA-damage-inducible 45 alpha |  |
| 73 | Iqgap1 | 0.389 | | IQ motif containing GTPase activating protein 1 |  |
| 74 | H2-Eb1 | 0.392 | | histocompatibility 2, class II antigen E beta |  |
| 75 | Epdr1 | 0.396 | | ependymin related protein 1 |  |
| 76 | Fam110a | 0.396 | | family with sequence similarity 110, member A , transcript variant 1 |  |
| 77 | Ifi27 | 0.397 | | interferon, alpha-inducible protein 27 |  |
| 78 | Olfml2b | 0.399 | | olfactomedin-like 2B |  |
| 79 | Tpst2 | 0.409 | | protein-tyrosine sulfotransferase 2 |  |
| 80 | Bhlhb2 | 0.416 | | basic helix-loop-helix domain containing, class B2 |  |
| 81 | H2-Ea | 0.416 | | histocompatibility 2, class II antigen E alpha |  |
| 82 | Prlr | 0.421 | | prolactin receptor |  |
| 83 | Gpc1 | 0.426 | | glypican 1 |  |
| 84 | Epb4.1l1 | 0.427 | | erythrocyte protein band 4.1-like 1 |  |
| 85 | Fcgr4 | 0.443 | | Fc receptor, IgG, low affinity IV |  |
| 86 | Igh-VJ558 | 0.447 | | PREDICTED: immunoglobulin heavy chain |  |
| 87 | Nrn1 | 0.455 | | neuritin 1 |  |
| 88 | Fam126a | 0.460 | | family with sequence similarity 126, member A |  |
| 89 | Nupr1 | 0.462 | | nuclear protein 1 |  |
| 90 | Prkca | 0.471 | | protein kinase C, alpha |  |
| 91 | Inmt | 0.473 | | indolethylamine N-methyltransferase |  |
| 92 | Prss35 | 0.474 | | protease, serine, 35 |  |
| 93 | Cox7a1 | 0.482 | | cytochrome c oxidase, subunit VIIa 1 |  |
| 94 | Rgcc | 0.491 | | RIKEN cDNA 1190002H23 gene |  |
| 95 | Tns3 | 0.496 | | tensin 3 |  |
| 96 | Atp6v0e2 | 0.498 | | ATPase, H+ transporting, lysosomal V0 subunit E2 |  |
| 97 | Fkbp5 | 0.506 | | FK506 binding protein 5 |  |
| 98 | Idh1 | 0.507 | | isocitrate dehydrogenase 1 , soluble |  |
| 99 | Nedd9 | 0.514 | | neural precursor cell expressed, developmentally down-regulated gene 9 |  |
| 100 | Frmd5 | 0.517 | | FERM domain containing 5 |  |
| 101 | Gas6 | 1.740 | | growth arrest specific 6 |  |
| 102 | Copz2 | 1.797 | | coatomer protein complex, subunit zeta 2 |  |
| 103 | Tcf3 | 1.840 | | transcription factor 3 , transcript variant 1 |  |
| 104 | Fxyd6 | 1.878 | | FXYD domain-containing ion transport regulator 6 |  |
| 105 | Itpkb | 1.917 | | inositol 1,4,5-trisphosphate 3-kinase B |  |
| 106 | Hba-a1 | 1.927 | | hemoglobin alpha, adult chain 1 |  |
| 107 | Mia1 | 1.974 | | melanoma inhibitory activity 1 |  |
| 108 | C79326 | 1.994 | | RIKEN cDNA 1110008P14 gene |  |
| 109 | Gstm6 | 2.014 | | glutathione S-transferase, mu 6 |  |
| 110 | Slc25a35 | 2.061 | | solute carrier family 25, member 35 |  |
| 111 | BC018465 | 2.086 | | cDNA sequence BC018465 |  |
| 112 | Rasl10a | 2.087 | | RAS-like, family 10, member A |  |
| 113 | Tmem100 | 2.105 | | transmembrane protein 100 |  |
| 114 | Stc1 | 2.135 | | stanniocalcin 1 |  |
| 115 | Sult1a1 | 2.157 | | sulfotransferase family 1A, phenol-preferring, member 1 |  |
| 116 | Kit | 2.167 | | kit oncogene |  |
| 117 | Kif1a | 2.239 | | kinesin family member 1A |  |
| 118 | Acta2 | 2.259 | | actin, alpha 2 |  |
| 119 | Gm1006 | 2.266 | | gene model 1006, |  |
| 120 | Fabp3 | 2.336 | | fatty acid binding protein 3, muscle and heart |  |
| 121 | Mt3 | 2.354 | | metallothionein 3 |  |
| 122 | AU021092 | 2.453 | | expressed sequence AU021092 |  |
| 123 | Slco2a1 | 2.456 | | solute carrier organic anion transporter family, member 2a1 |  |
| 124 | Tulp2 | 2.488 | | tubby-like protein 2 , transcript variant 1 |  |
| 125 | Dnahc2 | 2.500 | | dynein, axonemal, heavy chain 2 |  |
| 126 | Foxa3 | 2.542 | forkhead box A3 | | |
| 127 | Frag1 | 2.710 | FGF receptor activating protein 1 | | |
| 128 | Tgif1 | 2.730 | TGFB-induced factor homeobox 1 | | |
| 129 | C7 | 2.829 | complement component 7 | | |
| 130 | Cacna1d | 3.100 | | calcium channel, voltage-dependent, L type, alpha 1D subunit , transcript variant 2 |  |
| 131 | Spnb1 | 3.208 | | spectrin beta 1 |  |
| 132 | Insl3 | 3.302 | | insulin-like 3 |  |
| 133 | Mupcdh | 3.349 | | mucin-like protocadherin |  |
| 134 | Aqp5 | 3.823 | | aquaporin 5 |  |
| 135 | Spink4 | 4.115 | | serine peptidase inhibitor, Kazal type 4 |  |
| 136 | Lep | 4.457 | | leptin |  |
| 137 | Serpina5 | 5.067 | | serine (or cysteine) peptidase inhibitor, clade A, member 5 |  |
| 138 | Kcng4 | 5.129 | | potassium voltage-gated channel, subfamily G, member 4 |  |
| 139 | Serpina3c | 5.690 | | serine (or cysteine) peptidase inhibitor, clade A, member 3C |  |
| 140 | Gpha2 | 6.078 | | glycoprotein hormone alpha 2 |  |
| 141 | Gsta1 | 8.561 | | glutathione S-transferase, alpha 1 |  |
